# The Therapeutic Effects of Low-Frequency Electrical Stimulations Adjunct to Sodium Valproate on Seizure and Behaviors

**DOI:** 10.32598/bcn.9.10.280

**Published:** 2020-01-01

**Authors:** Raha Zalkhani, Ahmad Ali Moazedi, Zohreh Ghotbeddin, Mahdi Pourmahdi Borujeni

**Affiliations:** 1. Department of Biology, Faculty of Science, Shahid Chamran University of Ahvaz, Ahvaz, Iran.; 2. Department of Basic Sciences, Faculty of Veterinary Medicine, Shahid Chamran University of Ahvaz, Ahvaz, Iran.; 3. Department of Food Hygiene, Faculty of Veterinary Medicine, Shahid Chamran University of Ahvaz, Iran.

**Keywords:** Hippocampal kindling, Seizure, Sodium valproate, Emotional disturbances, Low-frequency stimulations

## Abstract

**Introduction:**

Consuming antidepressant medications induce several problems leading to the need for alternative agents for emotional disturbances. Antidepressant medications increase the seizure risk; thus, alternative treatments, like Antiepileptic Drugs (AED), might be useful for patients with epilepsy comorbid with a psychiatric disorder. The present study evaluated the behavioral effects of sodium valproate, a none effective dose in seizure treatment [100 mg/kg; Intraperitoneal (IP)] along with the application of Low-Frequency Stimulations (LFS) during CA1 hippocampal kindling.

**Methods:**

In total, 42 male rats were randomly divided into 6 groups, including control group with intact animals handled daily (I); sham group which was subjected to the surgical process, but received no real stimulation (II); saline-kindled Kindled group (S.kindled) which were stimulated daily with the following protocol: 3 strain of 50Hz monophasic pulses of 1ms duration applied 12 times a day with the threshold intensity at intervals of 10 minutes where saline was administrated 15 min before kindling stimulations (III); saline-kindled-LFS group (K4LFS) in which saline was injected 15 min before kindling stimulations and LFS was applied daily after the termination of kindling stimulation (IV); drug-kindle group (Drug100.kindled) that underwent rapid kindling procedure daily where sodium valproate (100 mg/kg) was administrated 15 min before kindling stimulations(V), and drug-kindled-LFS (Drug100.kindled.4LFS) group in which drug and LFS were administrated respectively before and after kindling stimulations (VI). The behavioral tests were assessed using elevated plus maze, open field, and forced swim tests.

**Results:**

The combination of sodium valproate (100 mg/kg) and LFS significantly decreased cumulative seizure severity compared with the kindle group. Thus, it provided a strong seizure suppressing effect. Additionally, sodium valproate and LFS increased the percentage of Open Arms (OAs) entries and the OAs exploration; they also decreased jumping from elevated plus maze test and rearing in open field test. Furthermore, there was no significant change in the OAs entries and OAs exploration percentages, jumping from apparatus, and rearing in open field in Drug100. Kindled, K4LFS, and Drug100.kindled.LFS groups, compared with the sham group. There was no significant difference in the latency to first immobility and the duration of immobility in K4LFS groups compared with the S. kindled group. In the drug-kindled group, the latency to first immobility significantly increased, and the duration of immobility decreased, compared with the S. kindled group. Besides, the latency to first immobility significantly increased, and the duration of immobility decreased in drug-kindled-LFS, compared to S. kindled group; however, the latency to first immobility was not significantly changed, compared to drug-kindled groups.

**Conclusion:**

Sodium valproate and LFS can modulate the function of the brain regions involved in emotional processing in epilepsy, as well as anxiety- and depressive-like behaviors. Such a combination could also decrease emotional disturbances induced by the kindling process.

## Highlights

- Hippocampal rapid kindling can induce several changes in the emotional behaviors of rats, including anxiety- and depressive-like behaviors.
- A combination of the sub effective dose of sodium valproate and LFS have a stronger anticonvulsant effect, compared to the single-use of each.
- A combination of the sub effective dose of sodium valproate and LFS significantly decreased emotional disturbance induced by kindling.

## Plain Language Summary

Applying antidepressant drugs generates several problems leading to the need for alternative agents for emotional disturbances. Since antidepressant drugs increase the seizure risk, alternative treatments, like antiepileptic drugs, might be useful for patients with epilepsy and a comorbid psychiatric disorder. Also, all seizures cannot be controlled by anti-epileptic drugs in refractory epilepsy. The present study evaluated the behavioral effects of the low dose valproate along with deep brain stimulations during epilepsy. The collected results suggested that deep brain stimulation could increase the effectiveness of sub effective doses of sodium valproate and decrease emotional disturbances induced by seizures.

## 1. Introduction

Emotional disturbances occur in various medical or neurological conditions (Grunze, 2008). Several comprehensive studies have demonstrated that patients with epilepsy suffer from high levels of anxiety and depression, compared to other neurological diseases (Volcy, 2004; Brandt, & Mula, 2016; Hamid, Ettinger, & Mula, 2011). Although anxiety disturbances might be more frequent in these patients, the main attention has been focused on depression. The prevalence of anxiety and depressive disorder has been respectively reported to be 19% and 11% in patients with temporal lobe epilepsy (Currie, Heathfield, Henson, & Scott,1971; Beyenburg, Mitchell, Schmidt, Elger, & Reuber, 2005).

Pharmacotherapy is dominantly performed for anxiety using benzodiazepine drugs (Von Moltke, & Greenblatt, 2003). These medications have been used as the main pharmacological treatment of anxiety over the last 4 decades. Consuming benzodiazepine drugs generates several problems leading to the need for alternative agents for anxiety conditions (Liability, 2005). Selective Serotonin Reuptake Inhibitors (SSRIs) can be beneficial in the treatment of approximately 50%–60% of patients with anxiety disorders; however, some patients cannot be successfully treated with SSRIs (Mula, Pini, & Cassano, 2007). Moreover, patients might become dependent on antidepressant drugs if using them for a long time (Suwanee, 2012). In addition, a majority of antidepressants reduce the seizure threshold, which in turn, may induce seizure attacks (Suwanee, 2012; Hill, Coupland, Morriss, Arthur, Moore, & Hippisley-Cox, 2015). For treating the depressive disorder, the initial treatment of depression includes medication and/or psychotherapy. Although it is estimated that 60%–70% of patients respond favorably to the initial treatment, other patients are considered to have chronic and refractory forms of depression. Therefore, it is important to explore alternative drug treatments, like Antiepileptic Drugs (AEDs) for uncontrolled depression, especially in patients with epilepsy (Hamani, & Nóbrega, 2010).

During the past three decades, AEDs have become important parts of the pharmacological treatment of various neurological disorders other than epilepsy (Spina, & Perugi, 2004; Hamid, Ettinger, & Mula, 2011). AEDs might have positive or negative psychotropic effects on a patient’s mood (Grunze, 2008; Brodie et al., 2016). Using AEDs, such as vigabatrin, phenobarbitone, tiagabine, and topiramate is associated with mood disturbances (Jackson, & Turkington, 2005). The adverse effects of these drugs on mood have been frequently reported; however, their positive effects were addressed for the first time in the 1960s (Grunze, 2008). Anti-anxiety effects of AEDs, like carbamazepine and lamotrigine, have been reported in various psychiatric disorders (Grunze, 2008).

Valproate is among the first AEDs, and its anticonvulsive characteristics were reported in 1963 (Chateauvieux, Morceau, Dicato, & Diederich, 2010). Recent studies have demonstrated that sodium valproate can produce obvious effects in treating various general and partial epilepsy (Chateauvieux, et al., 2010; Goldenberg, 2010). It can also serve as a reliable option to suppress generalized and focal seizures (Perucca, 2002).

Sodium valproate has been widely used as a psychotherapeutic drug and a novel method to treat the manic phase in numerous bipolar patients (Spina & Perugi, 2004). Several behavioral and clinical studies suggested that sodium valproate can simulate the anxiolytic effects of benzodiazepines (Mula, Pini, & Cassano, 2007; Jalilifar, Yadollahpour, Moazedi, & Ghotbeddin, 2017); however, most studies have only focused on healthy animal models. Thus, there is no reliable evidence to prove the effects of these drugs on the mood of patients with epilepsy.

Most information about the effects of anticonvulsant medicines on neurological diseases (except for epilepsy) is based on case reports and uncontrolled studies; therefore, they fail to determine the effectiveness and safety of these medications (Spina & Perugi, 2004).

Since epileptic seizures cause different alternations in the brain function, the validity of normal animal models for evaluating drugs’ effects is questionable (Löscher, 2011). Therefore, there is a requirement to use epileptic animals in exploring the effects of drugs. Epileptic animals are more susceptible to the behavioral changes following the administration of drugs (Jalilifar, et al., 2017).

Electrical kindling can be induced by daily electrical stimulation in limbic structures and is a beneficial option to investigate the process of epileptogenesis and anxiety disturbances in epileptic animals (Jalilifar, et al., 2017).

Brain stimulation has been reported as an efficient technique to suppress epileptic discharges and decrease seizure severity (Laxpati, Kasoff, & Gross, 2014; Zhang et al., 2012). Numerous studies investigated the mood-stabilizing and anxiolytic effects of valproate. However, data on the behavior-related effects of sodium valproate and LFS, as an effective combined therapy during hippocampal kindling in rats are scarce. Therefore, the present study aimed to evaluate the behavioral effects of 100 mg/kg Intraperitoneal (IP) of sodium valproate, a none-effective dose in seizure treatment, along with LFS application during CA1 hippocampal kindling.

## 2. Methods

All experiments and animal care procedures were approved by the Local Ethics Committee of the Shahid Chamran University of Ahvaz. This research completely coincides with the guide for the care and use of laboratory animals by the National Institutes of Health publication.

Adult male rats (Mean±SD weight: 200±20 g) were obtained from the animal house of Shahid Chamran University of Ahvaz. The animals were individually housed in transparent plastic cages with an ambient temperature of 23±2 oC, the humidity of 50%±5%, and a 12/12 h light/dark cycle (lights on from 7:00 AM).

Sodium valproate was purchased from Sigma and was dissolved in physiological saline (0.9% sodium chloride). The study animals received IP sodium valproate or saline at a volume of 1mL/kg body weight daily injections for 5 days.

All rats were anesthetized by a ketamine/xylazine mixture (100/10 mg/kg, IP) and fixed on the stereotaxic apparatus. One tripolar stainless steel electrode (bipolar for stimulating and monopole for recording) was positioned in the right hippocampus using Paxinos and Watson atlas (Paxinos & Watson, 2006) from bregma: anteroposterior: 2.5 mm; lateral:1.8 mm; vertical: 2.8 below the skull (Stoelting Co., USA). Besides, a monopolar electrode, used as ground and reference electrode, was attached to the rats’ skull with stainless steel crew. Electrodes were connected to pins and inserted into a socket and fixed in the skull with acrylic dental cement.

Electrical stimulations were applied with an electro-optic modulator device (Science beam Co., Tehran, Iran), connected to a computer for monitoring epileptiform ADs using the E-Probe software program.

At least one week after surgery, the After-Discharge (AD) threshold was evaluated using a 3 s train of 50Hz monophasic square pulses of 1ms duration. It was initially delivered at 30 *μ*A then increased in the steps of10 *μ*A with 10 minute intervals between current delivery until inducing ADs for at least 8s (Jalilifar, et al., 2017). From all rats implanted with electrodes, we could only use 42 rats because some exhibited no AD until 150 *μ*A. One day following AD threshold determination, the kindling procedure initiated, and the rats were electrically stimulated at the AD threshold 12 times a day.

In the present experiment, 42 rats were divided into 6 groups, as follows: control group in which intact animals were handled daily (I); sham group which was subjected to the surgical process, but received no real stimulation (II); saline-kindled group (s. kindled) which were stimulated daily with the following protocol: a 3 s train of 50Hz monophasic square pulses of 1ms duration with the threshold intensity, applied 12 times a day at 10-min intervals where saline was administrated 15 min before kindling stimulations (III), and saline-kindled-LFS group (K4LFS) in which saline was injected 15 min before kindling stimulations and LFS applied daily after termination of kindling stimulation (IV): LFS consisted of four packages at 5-min intervals. Drug-kindle group (Drug100.kindled) received sodium valproate (100 mg/kg) 15 min before kindling stimulations (V). In drug-kindled-LFS (Drug100.kindled.4LFS) group, drug and LFS were administrated respectively before and after kindling stimulations (VI). Each package contained 200 monophasic square wave pulses of 0.1-ms duration at 1 Hz.

The 100 mg/kg of sodium valproate is a non-effective dose in seizure treatment. The present study aimed to investigate the effect of this dose of valproate combined with applying LFS immediately after the termination of kindling stimulation in seizure severity, as well as anxiety- and depressive-like behaviors. The seizure severity of the kindling process was calculated according to the Racine scale (score 1: facial clonus, immobility, and rigid posture; score 2: facial movement and head nodding; score3: forelimb clonus; score 4: rearing with the tonic extension of forelimbs; & score 5: the loss of righting reflex and balance) (Kalynchuk, 2000). The kindling stimulations were administered until the emergence of stage 5 seizures (it took 5 days). Accordingly, the animals were treated with sodium valproate or LFS for 5 days. Seizure severity was evaluated through the observation of the animal’s behavior immediately after kindling stimulation. Since all animals were stimulated 12 times per day, cumulative seizure severities were measured (as the summation of seizure severity after daily stimulations). Anxiety- and depressive-like behaviors were determined respectively by the Elevated Plus Maze (EPM) and open field tests on the 6th day of the experiment and Forced Swim Test (FST) on the following day.

The EPM test has been verified to analyze anxiety-related behaviors in rodents. The instrument was a plus shape and had two open arms opposite each other and two Close Arms (CAS). The arms were elevated 70 cm above the floor. During the experiment, each rat was placed in the central platform of EPM facing an open arm and was allowed to freely explore the maze for 5 min (Sestakova, Puzserova, Kluknavsky & Bernatova, 2013).

The numbers of entries and time exploration in each arm and the percentage of entries and time explorations into the OAs were calculated. The percentage of OAs entries was calculated as the number of entries into the OAs/number of OAs + CA entries × 100. The percentage of OAs exploration was equal to the time spent in the open arms / the time spent in both arms of the maze × 100 (Rodgers & Dalvi, 1997). Ratio time and entry determined the anxiety level in the EPM. The smaller the time or entry ratio, the greater the anxiety in rats. After each trial, the set up was completely cleaned with an ethanol solution to remove any olfactory cue (Andreatini, & Bacellar,1999; Godlevsky, Muratova, Kresyun, Van Luijtelaar, & Coenen, 2014).

### 2.1. Open field test

An open field box consists of a white floor and 4 transparent Plexiglas-walls (size: 45×45×40 cm). Rearing frequency (the number of times the animal stood on its hind legs) was measured as the behavioral element. The rearing has also been regarded as an exploratory behavior aspect in some studies (Rodgers & Dalvi, 1997; Godlevsky, et al., 2014); however, some studies suggested that anxiolytic agents decrease the number of rearing. Each rat was placed in the center of the box and allowed to explore the open field for 5 min. After each trial, the set up was cleaned thoroughly with an ethanol solution to remove any olfactory cue (Ago, Takahashi, Nakamura, Hashimoto, Baba, & Matsuda, 2007).

Forced Swim Test (FST) is among the most commonly used assays to monitor depressive-related behaviors in rodents. The FST provides a situation to evaluate the animal’s ability to escape from an inescapable stressful condition; a higher immobility duration indicates depression-like behavior (Shumake, & Gonzalez-Lima, 2003).

The test was performed between 9:00 AM and 1:00 PM. The FST consisted of two sessions conducted 24 h apart. The first session is the pre-test stage (it takes 15 min), and the second session is the test stage (5min). The cylinder was filled with adequate water (25°C, depth=47 cm) to ensure that the rats’ hind-paws could not touch the cylinder’s bottom. The study rats were individually forced to swim in a plastic cylinder. Immobility duration, including passive swimming or floating in the water without struggling, was measured, and they were then placed back in drying cage. After each, the container was refilled with fresh water to avoid any influence on the next rat (Godlevsky, et al., 2014; Slattery, & Cryan, 2012).

The obtained data were analyzed using SPSS. The collected data were represented as the mean±Standard Error of Mean (SEM). A one-way Analysis of Variance (ANOVA), followed by a Tukey posthoc test was performed to compare the changes in the percentage of entries into the OAs, the percentage of OAs exploration in EPM, the rearing in the open field, the latency to first immobility, and the immobility duration in FST. Moreover, cumulative seizure severity during the kindling process was compared using repeated-measures ANOVA. The analyses were performed two-sided, and the significance level was adjusted at P≤0.05.

## 3. Results

**Figure 1.**
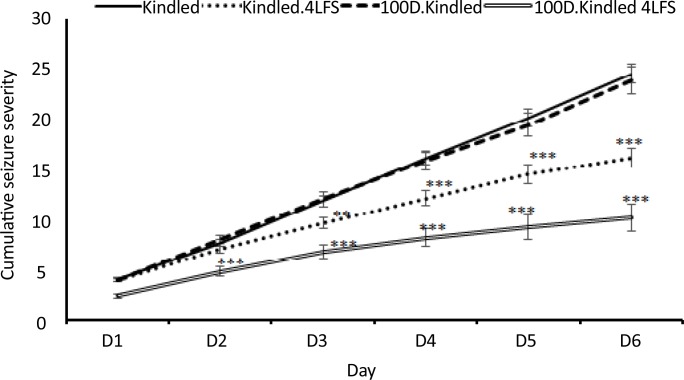
The effects of sodium valproate and LFS on cumulative seizure severity ^*^P≤0.05; ^**^P≤0.01; ^***^ P≤0.001; compared to the sham group; Values are presented as mean±SEM.

The effects of sodium valproate (100 mg/kg) and LFS, respectively, before and after kindling stimulations on seizures induced by rapid hippocampal kindling were measured. Figure 1 shows the effect of 100 mg/kg IP of sodium valproate injection on the cumulative seizure severity. This dose did not affect the seizure treatment (Figure 1). Our findings also indicated that LFS decreased the cumulative seizure severity during rapid hippocampal kindling (P=0.001). However, when these two treatments were combined, the cumulative seizure severity significantly decreased, compared with s. kindled group. This process indicated a stronger anticonvulsant effect, in comparison with using sodium valproate or LFS alone (Figure 1).

There was no significant difference in the percentage of entries and exploration of OAs in the elevated plus-maze, the rearing in the open field, latency to first immobility, and immobility duration in the FST between the control and sham groups. The achieved results also revealed that electrode implantation could not affect anxiety- and depressive-like behaviors.

**Figure 2.**
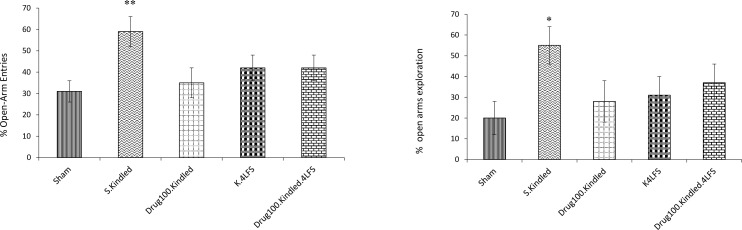
The effects of sodium valproate and LFS on anxiety-like behaviors in the elevated plus-maze ^*^P≤0.05; ^**^P≤0.01; ^***^ P≤0.001; Compared to the sham group; values are presented as Mean±SEM.

**Figure 3.**
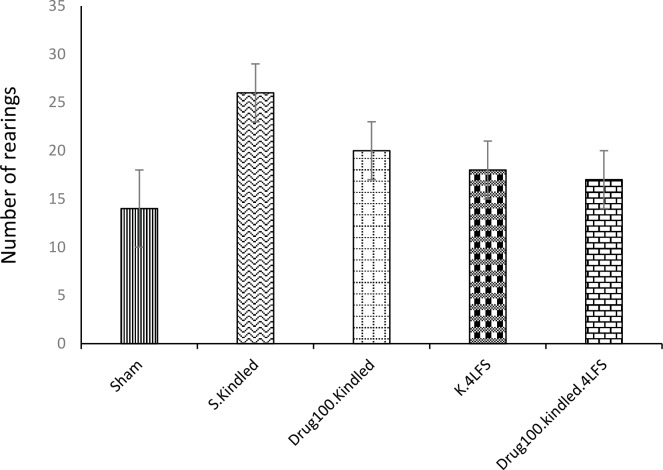
The effects of sodium valproate and LFS on anxiety-like behaviors in the open field test ^*^P≤0.05; ^**^P≤0.01; ^***^ P≤0.001; compared to the sham group; values are presented as mean±SEM.

The percentages of OAs entries (P=0.005) and explorations (P=0.02) significantly increased in the S. kindled group, compared to the sham group in the elevated plus-maze (Figure 2). Similarly, the jumping from the elevated plus-maze significantly increased in them, compared to the sham group (Table 1). The number of rearing in the open field significantly increased in s. kindled rats, in comparison with the sham group (P=0.006) (Figure 3).

**Table 1.** The number of jumps from the elevated plus-maze test

**Group**	**Number of Jumps From the Elevated Plusmaze Test**
Control	0
Sham	0
Saline. Kindled	10
Kindled. LFS	0
100drug.Kindled	0
Drug100.Kindled.LFS	0

Besides, no significant difference was observed in the percentage of OAs entries and explorations between the drug-kindled, K4LFS, drug-kindled-LFS, and sham groups (Figure 2). Applying LFS, pharmacotherapy, and a combination of these treatments decreased the number of jumps from the EPM, compared to the kindled group (Table 1). The number of rearing was not significantly increased in the drug-kindled, K4LFS, drug-kindled-LFS groups, compared with the sham group (Figure 3). These findings suggested no significant difference in the anxiety level in these groups, compared with the sham group; thus, the medication and LFS decreased kindling-induced anxiety in the experimental groups.

**Figure 4.**
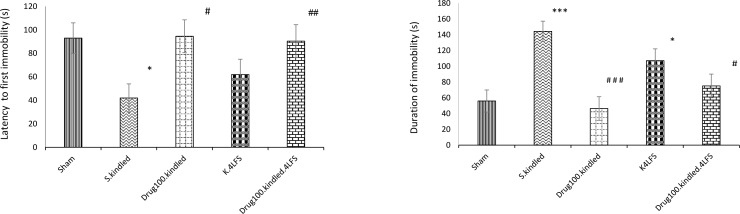
The effects of sodium valproate and LFS on depressive-like behaviors ^*^P≤0.05; ^**^P≤0.01; ^***^ P≤0.001; Compared to the sham and #Compared to the S. Kindled group; Values are indicated as Mean±SEM.

In the S. kindled group, the latency to first immobility decreased, compared with the sham group (P=0.013). The immobility duration in the FST significantly increased in the s. kindled group, compared with the sham group (P=0.000) (Figure 4).

In the drug-kindled group, the latency to first immobility increased, compared with s. kindled group (P=0.022), and the change was not significant, compared to the sham group. The immobility duration in the drug-kindled group significantly decreased, compared with the s. kindled group (P=0.000); however, it was not significantly changed in comparison with the sham group (Figure 4).

In the K4LFS group, the latency to first immobility increased; however, this change was not significant compared with the s. kindled group. Moreover, the latency to first immobility in the K4LFS was not significantly changed in comparison with the sham group. The immobility duration decreased in the K4LFS group, compared with the kindled group; however, it was not significant. This duration significantly changed in the K4LFS group, in comparison with the sham group (P=0.059) (Figure 4).

In the drug-kindled-LFS group, the latency to first immobility was not significantly changed in comparison with the sham and drug-kindled groups. Besides, there were significant alterations in latency to first immobility (P=0.056) and the immobility duration (P=0.010) in the drug-kindled-LFS group, compared to the s. kindled group (Figure 4).

## 4. Discussion

The obtained data demonstrated that LFS could increase the effectiveness of the sub-effective doses of sodium valproate. Our results were consistent with those of some studies reporting that the electrical stimulation of the brain combined with antiepileptics acting on the Gamma-Aminobutyric Acid (GABA) system can provide better control of epileptic seizures (Asgari, Semnanian, Atapour, Shojaei, Moradi, & Mirnajafi-Zadeh, 2014). Asgari et al., suggested that LFS adjunct to barbiturates increased the efficacy of each of these treatments and reduced seizures in amygdala-kindled rats (Asgari et al., 2014). Moreover, Cuellar-Herrera et al., reported that implementing High-Frequency Stimulation (HFS) combined with antiepileptic drugs, including diazepam, phenobarbital, and gabapentin increased the HFS-induced anticonvulsant effects (Cuellar-Herrera, Pena, Alcantara-Gonzalez, Neri-Bazan, & Rocha, 2010). A clinical study suggested that applying HFS to the thalamus combined with the drugs improved deep brain stimulation anticonvulsant actions (Kerrigan et al., 2006). However, some antiepileptics, like phenytoin, i.e. a drug that inhibits voltage-dependent sodium currents, combined with electrical stimulation, avoids its protective effects (Asgari et al., 2014; Cuellar-Herrera et al., 2010). It seems the association of LFS with a sub-effective dose of sodium valproate increased the effects of the GABA system, to provide better protection against seizures (Figure 1).

The second part of the study evaluated the effects of sodium valproate and LFS on animal behaviors in the kindling process. The relevant results indicated that rapid hippocampal kindling could induce several changes in the emotional behaviors of rats, including increasing the percentage of OAs entries and explorations, jumping, and rearing (Figures 2 & 3). The increased percentage of OAs entries and OAs exploration are generally used to analyze anxiolytic drug effects; however, all rats in the kindled group jumped from the elevated plus maze, and rearing in the open field increased. Accordingly, we concluded that the level of fear in rats might have increased to the extent that they searched for routes to escape from the apparatus. This finding was consistent with those of Lisa E.Kalynchuk et al., reporting kindling generated behavioral changes and had an anxiogenic effect (Kalynchuk, Pinel, Treit, & Kippin, 1997; Kalynchuk, Pinel, & Treit,1998).

The achieved results also indicated that using sodium valproate (100 mg/kg) significantly reduced anxiety-like behavior in the studied rats in the elevated plus-maze and open field tests. Several studies reported the effectiveness of anticonvulsant drugs in the treatment of psychiatric disorders. Our findings were consistent with those of Lang and De Angelis (2003) and De Angelis (1992), in which the anxiolytic-like effects of sodium valproate in the mirrored chamber test and the light/dark aversion test were respectively analyzed. In addition, Kinrys et al., demonstrated that sodium valproate could be an efficient option to treat patients with a social anxiety disorder (Kinrys & Wygant, 2005). Besides, AEDs, like valproate, can be used in patients resistant to SSRI via increasing the efficiency of GABA (Van Ameringen, Mancini, Pipe, & Bennett, 2004; Brambilla, Perez, Barale, Schettini, & Soares, 2003). There is a common pathophysiological mechanism for anxiety and epilepsy attacks. AEDs can be beneficial in anxiety disorders since they reduce seizures through decreasing the excessive outburst from epileptic neurons, reducing anxiety symptoms, and decreasing neuronal activation within fear circuits (Jones, Salzberg, Kumar, Couper, Morris, & O’brien, 2008).

We also found that rapid hippocampal kindling can increase immobility (despair) in the FST. This result was consistent with that of Mazarati et al. (2009), who reported rapid kindling increased immobility in the FST and the loss of taste preference towards calorie-free saccharin in immature kindled rats. They concluded that brain changes induced by kindling were accompanied by the development of depressive behavior.

Our data also suggested that the latency to first immobility increased while the immobility duration decreased in Drug100. Kindled, compared with the kindled group. Thus, sodium valproate had a significant antidepressant effect. This finding was in line with that of Qiu HM et al., suggesting that valproate use decreased the immobility time in depressive rats. They reported that the antidepressant effect of valproate is related to improving the hypothalamic-pituitary-adrenal axis function, elevating the BDNF, and 5-HTT expression, as well as decreasing the MAO-A and IDO expression of the hippocampus in depressive rats. Valproate decreased catecholamine levels in the brain, i.e. associated with emotional behaviors (Qiu, Yang, Liu, Fei, Hu, & Zhou, 2014; Qiu, Yang, Jiang, Hu, Liu, & Zhou, 2015). Carlson et al. (2006) suggested that valproate reduces brain infarction and neurological deficits through inhibiting caspase-3 activation and inducing chaperone proteins. Kindling adversely affects normal serotonergic transmission and produces excessive glucocorticoids; thus, contributing to aggravated fear and depressed mood (Baf, Subhash, Lakshmana, & Rao,1994; Wilson, McLaughlin, Ebenezer, Nair, & Francis, 2014; Maes, Calabrese, Jayathilake, & Meltzer,1997).

The obtained data revealed no significant difference in the percentage of entries and time explorations, jumping from apparatus, and the rearing between K4LFS, drug-kindled-LFS, and sham groups. Besides, in these groups, the latency to first immobility increased while the immobility duration decreased (P=0.01), compared to the s. kindled group. Therefore, the application of LFS alone or with sodium valproate reduced anxiety- and depressive-like behaviors induced by kindling.

Several studies suggested that electrical brain stimulation reduces seizure frequency in epileptic patients and animal models (Jalilifar et al., 2017; Asgari et al., 2014; Cuellar-Herrera et al., 2010; Esmaeilpour, Sheibani, Shabani, Mirnajafi-Zadeh, & Akbarnejad, 2018); however, there is a lack of an appropriate study about the effectiveness of combined therapy of antiepileptic drugs with LFS; thus, future studies are required to examine it (Asgari et al., 2014; Cuellar-Herrera et al., 2010). The archived results reported the effects of LFS combined with sodium valproate on behaviors during rapid kindling. In conclusion, the combination of sodium valproate (100 mg/kg) and LFS provides a strong seizure suppressing effect and modulates emotional disturbances.

## 5. Conclusion

Sodium valproate and LFS can modulate the function of the brain regions involved in emotional processing in epilepsy, as well as anxiety- and depressive-like behaviors. They could also decrease emotional disturbances induced by the kindling process.
